# Development of Tandem Mass Tag Labeling Method for Lipid Molecules Containing Carboxy and Phosphate Groups, and Their Stability in Human Serum

**DOI:** 10.3390/metabo11010019

**Published:** 2020-12-30

**Authors:** Suzumi M. Tokuoka, Yoshihiro Kita, Masaya Sato, Takao Shimizu, Yutaka Yatomi, Yoshiya Oda

**Affiliations:** 1 Department of Lipidomics, Graduate School of Medicine, The University of Tokyo, Hongo 7-3-1, Bunkyo-ku, Tokyo 113-8654, Japan; stokuoka@m.u-tokyo.ac.jp (S.M.T.); kita@m.u-tokyo.ac.jp (Y.K.); tshimizu@ri.ncgm.go.jp (T.S.); 2 Department of Clinical Laboratory Medicine, Graduate School of Medicine, The University of Tokyo, Hongo 7-3-1, Bunkyo-ku, Tokyo 113-8654, Japan; masayasato0407@gmail.com (M.S.); yatomiy-lab@h.u-tokyo.ac.jp (Y.Y.); 3 Department of Lipid Signaling, National Center for Global Health and Medicine, Toyama 1-21-1, Shinjuku-ku, Tokyo 162-8655, Japan

**Keywords:** high-throughput, relative quantitation, lipids with carboxy groups, lipids with phosphate groups

## Abstract

In clinical lipidomics, it is a challenge to measure a large number of samples and to reproduce the quantitative results. We expanded the range of application of the tandem mass tag (TMT) method, which is widely used in proteomics, to lipidomic fields. There are various types of lipid molecule, for example, eicosanoids have a carboxyl group and phosphatidic acid has a phosphate group. We modified these functional groups simultaneously with TMT. This approach allows for a single analysis by mixing six samples and using one of the six samples as a bridging sample; the quantitative data can be easily normalized even if the number of measurements increases. To accommodate a large number of samples, we utilize a pooled serum sample of 300 individuals as a bridging sample. The stability of these lipid molecules in serum was examined as an analytical validation for the simultaneous TMT labeling. It was found that the stability of these lipid molecules in serum differs greatly depending on the lipid species. These findings reaffirmed the importance of proper sample preparation and storage to obtain reliable data. The TMT labeling method is expected to be a useful method for lipidomics with high-throughput and reliable reproducibility.

## 1. Introduction

Lipid molecules are involved in many physiological processes and are thought to be associated with a variety of diseases [1,2,3]. Omega-3 polyunsaturated fatty acids (PUFAs), including eicosapentaenoic acid (EPA) and docosahexaenoic acid (DHA), might be beneficial for human health [4,5,6]. Phosphatidic acid (PA) is the simplest glycerophospholipid present in cells. Recently, PA has also been thought to be a second messenger in signal transduction [7,8,9]; it is converted to diacylglycerol (DAG) by phosphohydrolases and is responsible for protein kinase C activation. In addition, PA is rapidly converted to lysophosphatidic acid (LPA) [10], a bioactive phospholipid present in various tissues, by phospholipases. Quantitative analysis of these lipids in clinical samples is crucial for understanding the function of bioactive lipids in physiology and various diseases. The most commonly used assay methods are gas chromatography (GC) or high-performance liquid chromatography (HPLC) coupled to mass spectrometry (MS). The development of LC/MS lipidomic profiling has enabled the more comprehensive analysis of various types of lipid molecule, and the importance of internal standards (ISs) was recognized soon after the advent of quantitative analysis by MS. In particular, the use of stable isotope-labeled lipids as ISs has evolved with the development of MS. However, it is not practical to prepare many stable isotope-labeled lipids for each target molecule. Furthermore, it is not easy to prepare the amount of ISs that need to be added to the sample in accordance with the concentration of the target molecule [11]. To solve this problem, Oda et al. developed a metabolic labeling method using stable isotope elements based on proteomics [12]. With this technique, accurate relative quantification was achieved without ISs; however, this method is limited to cells that can be cultured and is extremely difficult to apply to clinical specimens. Isotope-coded affinity tags [13] and isotope-coded protein labels [14] have been developed for this purpose. Although the relative quantification of proteins with reasonable accuracy became available with these methodologies, these methods require double target MS for measurements because of the isotope used for labeling. In addition, when relatively inexpensive deuterium is used for labeling, the target molecule is sometimes separated from the IS by HPLC or capillary GC, which results in differences in the ionization efficiency (ionization suppression) of the MS and thus causes quantitative discrepancies [15,16]. Therefore, methods such as Isobaric Tag for Relative and Absolute Quantitation [17] and tandem mass tag (TMT) [18] have been developed to quantify the target molecule using MS/MS product ions. In these methods, the precursor ion in MS is a common *m*/*z*, but the product ion *m*/*z* is different between the compared samples and, thus, the relative quantitation is performed by MS/MS. As the tags were developed for multiplexing, the number of measurements can be reduced because several compared samples are mixed together for measurement, which allows sample throughput to be increased. In the sixplex TMT method, six samples are measured in a single run. This multiplex method has advantages in not only throughput, but also relative quantification. Each TMT- derivatized molecule in the six samples elutes at exactly the same time, allowing the matrix effect among these six samples is exactly the same. Many papers in the field of proteomics have demonstrated the good reliability of relative quantification. Isobaric tag approaches in lipidomics [19] and metabolomics [20] for the compounds with amino groups have been used, the TMT labeling method for carboxy groups has been developed as a functional group other than amino groups [21], and we have previously conducted the quantitative analysis of free fatty acids using approximately 200 human plasma samples [22]. To take advantage of relative quantification by TMT labelling, we propose a strategy for using a common quality control (QC) sample each time for one of six sample in sixplex measurement, thereby allowing for comparisons of large number of sample sets as well as different datasets. In the present study, we developed a method for the simultaneous quantitative determination for carboxy-containing lipids, not only free fatty acids, but also their related metabolites, such as eicosanoids, as well as phosphate groups containing lipids, PAs, and looked at their stability in serum samples.

## 2. Results and Discussion

### 2.1. Lipid Extraction

**Figure 1:**
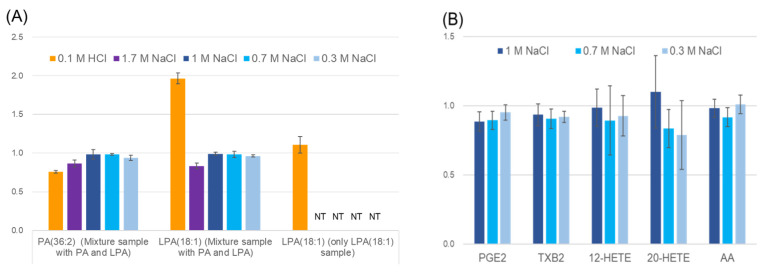
Examination of 1-buthanol extraction conditions. The *Y*-axis is the ratio of the peak area after extraction to the peak area without the extraction process. Error bars represent standard deviations. (**A**) Extraction efficiencies of phosphatidic acid (PA) and lysophosphatidic acid (LPA). The left and middle panels represent the recovery rate for 1-butanol extraction with each acidic or high salt condition. Mixture samples of 44 ng of LPA (18:1) and 50 ng of PA 18:1/18:1 (PA (36:2) in a methanol stock solution were put in test tubes. After evaporation, 330 μL of phosphate buffered saline (PBS) was added, followed by 1-buthanol extraction with various conditions (acidic or salt). Three replicates were tested for extraction. (**B**) Extraction efficiencies of several eicosanoids. The recovery of eicosanoids and arachidonic acid (AA) by 1-butanol extraction with each salt condition is shown. A mixture of 5.1 ng of each 12-hydroxyeicosatetraenoic acid (12-HETE), 20-hydroxyeicosatetraenoic acid (20-HETE), prostaglandin E_2_ (PGE_2_), thromboxane B_2_ (TXB_2_), and AA were put in the test tube. After evaporation, 30 μL of PBS was added followed by 1-buthanol extraction with the indicated conditions. Three replicates were tested for the extraction.

Methanol extraction method is simple and has a good recovery rates for medium-polarity lipids but do not remove many impurities that compete with chemical modification reactions (see below). In the blood, there are many polar metabolites with carboxyl and phosphate groups, such as organic acids, nucleic acids and amino acids. In the reaction, these molecules would compete with our TMT derivatization for lipids; therefore, the removal of such molecules by some lipid extraction methods is desirable. The extraction efficiency of LPA and hydroxylated PUFA, which have relatively high polarity, is poor in the Bligh–Dyer extraction method [23,24]. For practical reasons, we decided to perform 1-butanol extraction with the target organic layer on top and water layer at the bottom. Such lipid extraction methods with 1-buthanol have been used for a long time. Changing the sample pH is used to increase the extraction efficiency [23]; however, some lipids are unstable under both acidic and weakly alkaline conditions. For example, lysophosphatidylcholine (LPC), which exists in biological fluids, is degraded to LPA by acid, leading to an overestimation of LPA [25,26]. Considering the buffering capacity of the serum, we added 1 M HCl to the serum at a final concentration of 0.1 M HCl and confirmed that the pH of the serum was 3 to 4. Then, the extraction was performed with 1-buthanol. As shown in Figure 1A, the yield of lyso-phosphatidic acid (18:1) (LPA (18:1)) was sufficient under acidic conditions; however, when we tested the sample of mixtures of diacylphosphatidic acid (18:1/18:1) (PA (36:2) and LPA (18:1), approximately twice as much LPA was recovered compared to the input, and the recovery of PA (36:2) was inadequate. This might have been due to the fact that LPA (18:1) was observed as a result of the degradation product of PA (36:2) in the starting material. To improve the extraction efficiency, sodium chloride was added to the sample instead of using acidic conditions. As shown in Figure 1, an increase in LPA due to degraded PA was not observed, and the yield was sufficient. We also confirmed that arachidonic acid and several eicosanoids also showed good recovery rates with this butanol extraction method using sodium chloride (Figure 1B).

### 2.2. Tandem Mass Tag (TMT) Derivatization

**Figure 2:**
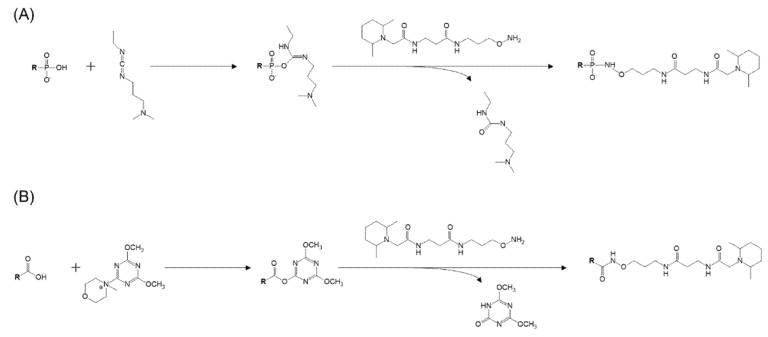
Chemical reaction schemes. (**A**) Tandem mass tag (TMT) derivatization for the phosphate group. (**B**) TMT derivatization for the carboxy group.

**Figure 3:**
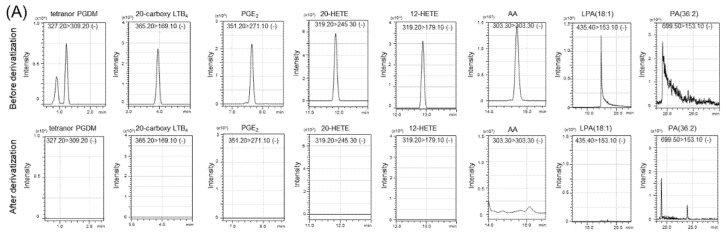

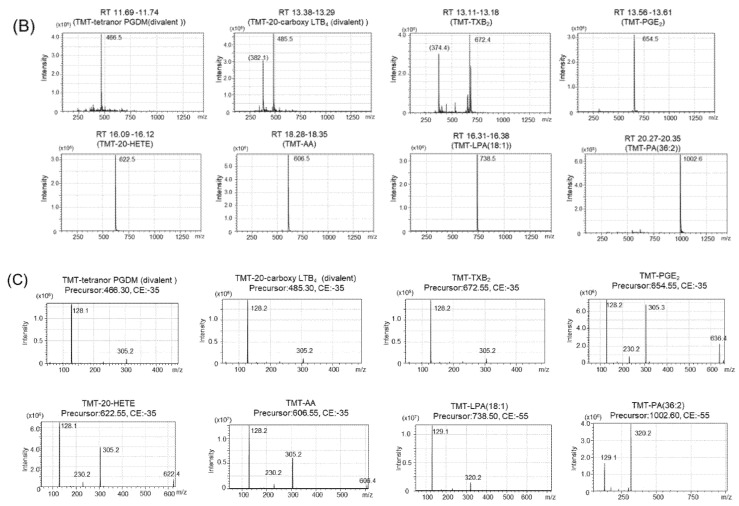
Derivatization of eicosanoids, fatty acids, lysophosphatidic acid and diacylphosphatidic acid. (**A**) Examples of chromatograms for input standards before (upper) and after (lower) derivatization with serum. Input signal compounds without derivatization was almost undetectable after derivatization. Selective reaction monitoring (SRM) transitions were shown at the top in each chromatogram as Q1 > Q3 (polarity). Injection volume was 5 μL and the concentration of compounds were 0.85 ng/μL each for all targets except for LPA (18:1) at 17 ng/μL and PA (36:2) at 2 ng/μL. (**B**) Representative precursor ion mass spectra obtained by tandem mass tag (TMT) reporter ion-specific precursor ion scanning analysis. Injection volume was 5 μL and the concentration of standards for derivatization was 42.5 pg /μL each for all targets except for LPA(18:1) at 17 ng/μL and PA(36:2) at 2 ng/μL. Eicosanoids and AA was derivatized with aminoxy TMT-128 and precursor ion scanning for 128.15 was performed. LPA (18:1) and PA (36:2) was derivatized with aminoxy TMT-129 and precursor ion scanning for 129.15 was performed. Positive ion mode and collision energy at −45 eV were used. Only the mass spectra at the retention time indicated for the target was shown. 11,15-Dioxo-9α-hydroxy-2,3,4,5-tetranorprostan-1,20-dioic acid (Tetranor PGDM) and 20-carboxy Leukotriene B_4_ (LTB4) have two carboxy groups to be derivatized, and divalent ions were detected. Note that RT 13.11-13.18 and RT 13.38-13.29 showed unexpected *m*/*z* (in parenthesis). Mass chromatograms for those ions were not identical to TMT-TXB2 or TMT-20-carboxy LTB4. (**C**) Representative product ion mass spectra obtained by product ion scans of derivatized compounds. Concentration of compounds, used TMT reporters and injection volume were same as (**B**). Precursor ion (*m*/*z*) and collision energy (eV) were indicated.

1-Ethyl-3-(3-dimethylaminopropyl)carbodiimide (EDC) is an easy-to-use and relatively inexpensive cross-linker that is soluble in water and methanol, forming active ester intermediates with phosphate and carboxyl groups. It then undergoes nucleophilic substitution in the presence of strong nucleophiles, such as primary amines. EDC cross-linking is commonly used for the bioconjugation of DNA and nucleic acids or for binding DNA to microplate wells under neutral conditions as one-pot reaction methods [27]. For liquid-phase coupling, the reaction with EDC is almost complete (95%) within 16 h at room temperature [28]. When we used the EDC reaction to label PAs with aminoxyTMT, the reaction proceeded without any problems. Since this reaction also proceeded for the carboxy group, unreacted free fatty acids were observed when the fatty acids and PA reacted simultaneously. When EDC was added to the one-pot reaction solution containing 4-(4,6-dimethoxy-1,3,5-triazin-2-yl)-4-methylmorpholinium chloride (DMTMM) for the TMT labeling of free fatty acids, which we have already reported [22], no unreacted PAs or fatty acids were detected, and the progress of TMT formation based on PAs and free fatty acids was observed. When there was no EDC reagent and only DMTMM was used, the TMT derivatization reaction proceeded to free fatty acids, but it did not react with PA. Figure 2 shows these chemical reaction schemes for phosphor-lipids and carboxy-lipids. Since the modification of phosphate groups by EDC cross-linking is widely used, it may be possible to label various phosphate groups with TMT. However, phosphatidylcholine, phosphatidylethanolamine, and their lyso-forms could not be labeled with TMT, probably because two of the oxygen atoms of the phosphorus atom are bonded to carbons. Input standard compounds without derivatization was not detectable indicating derivatize efficiency was sufficient (Figure 3A). Representative spectra obtained by TMT reporter ion-specific precursor ion scanning analysis and product ion scan spectra of derivatized PA, Lyso-PA(LPA), arachidonic acid and eicosanoid standards are shown in Figure 3B,C. 

### 2.3. Reproducibility

Next, the reproducibility of TMT labeling was confirmed. Authentic standards of PA, LPA, and eicosanoid-related fatty acids were added to phosphate buffer saline (PBS) or human pooled serum at 2-, 4-, 8-, and 16-fold dilutions, and 1-buthanol extraction was performed in 0.7 M sodium chloride. The repeatability of TMT derivatization in the presence or absence of serum was satisfactory, with a coefficient of variation ranging from 1.8% to 28.4%, as shown in Table 1. The reproducibility was slightly better in serum than in phosphate buffer. The reason for this might be that lipids are difficult to mix with PBS and that adsorption to the container is a concern; therefore, experiments with lipids in the presence of serum could have better reproducibility. The relative linearity between the area value of TMT-derived targets and the sample dilution factor of the TMT-labeled lipids ranged from 0.940 to 0.999 as a correlation coefficient, regardless of the presence or absence of serum. From the results of assay validation shown in Table 1, there were no degradation reactions or other factors that would affect the reproducibility. Our reaction conditions are very mild in terms of pH and temperature, however, there may be slightly unstable lipid molecules during derivatization, and they do not affect the relative quantification because they are derivatized together and normalized by the bridging sample measured simultaneously.

### 2.4. Stability of Phosphatidic Acid (PA) in Serum

**Figure 4:**
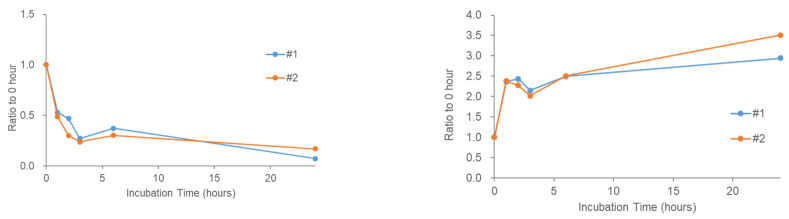
Stability of phosphatidic acid (PA) (36:2) in human serum during 37 °C incubation. A standard stock of PA (18:1/18:1) was dissolved in serum and incubated for the indicated time. Two replicate samples with 5.1 ng of PA in 30 µL pooled serum were tested. (**left**) PA (36:2) and (**right**) LPA (18:1) were analyzed using liquid chromatography/mass spectrometry (LC/MS) after lipid extraction. The *X*-axis shows incubation time (h) and the *Y*-axis shows values relative values to those at 0 h. Two replicate sample results (#1 and #2) are shown as lines.

**Figure 5:**
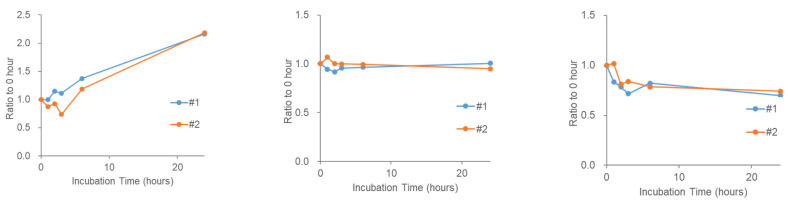
Stability of endogenous lysophospholipids and diacylglycerol in human serum during 37 °C incubation. Pooled serum was incubated for the indicated time and (**left**) lysophosphatidylcholine (LPC) 18:1, (**middle**) lysophosphatidylethanolamine (LPE) 18:1, and (**right**) diacylglycerol (DAG) 18:1/18:1 were analyzed using LC/MS after lipid extraction. The *X*-axis shows incubation time and values are shown on the *y*-axis relative to those at 0 h. Two replicate sample results are shown as lines.

During reproducibility experiments using serum samples, we were unable to detect any endogenous PAs in the serum. Although the sensitivity of this study depends partly on the MS performance and HPLC conditions, the stability of PA in the serum was investigated by adding a PA standard to the human pooled serum. We observed a rapid decrease in PA added to the serum (Figure 4 (left)) and an increase in LPA instead (Figure 4 (right)). This is consistent with the fact that serum LPA levels are already known to increase during serum incubation [25,29]. To examine the stability of several other lipid signaling molecules that were not labeled with TMT, they were measured using the same pooled serum sample (serum supplemented with PA). LPC is thought to be a source of LPA via action of the extracellular enzyme autotaxin, and the increase in LPC during serum incubation has also been reported [28]. As expected, LPC (18:1) tended to increase in the serum (Figure 5 (left)). In contrast, lysophosphatidylethanolamine (LPE) (18:1) did not change significantly (Figure 5 (middle)) and DAG (18:1/18:1) appeared to decrease slightly in the serum (Figure 5 (right)). These results suggest that the stability of the bioactive lipids in serum varies depending on the molecular species.

### 2.5. Stability of Fatty Acids and Their Metabolites in Serum

We collected 172 serum samples from patients over 62 years of age with five diseases (Alzheimer’s disease, Parkinson’s disease, depression, schizophrenia, and stroke) from the University of Tokyo Hospital (Table 2).

**Figure 6:**
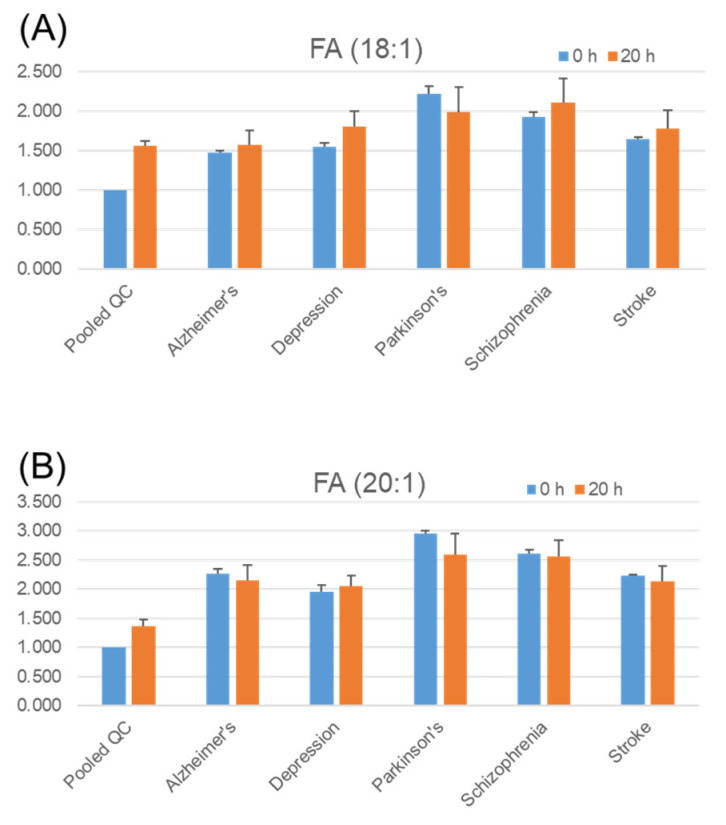

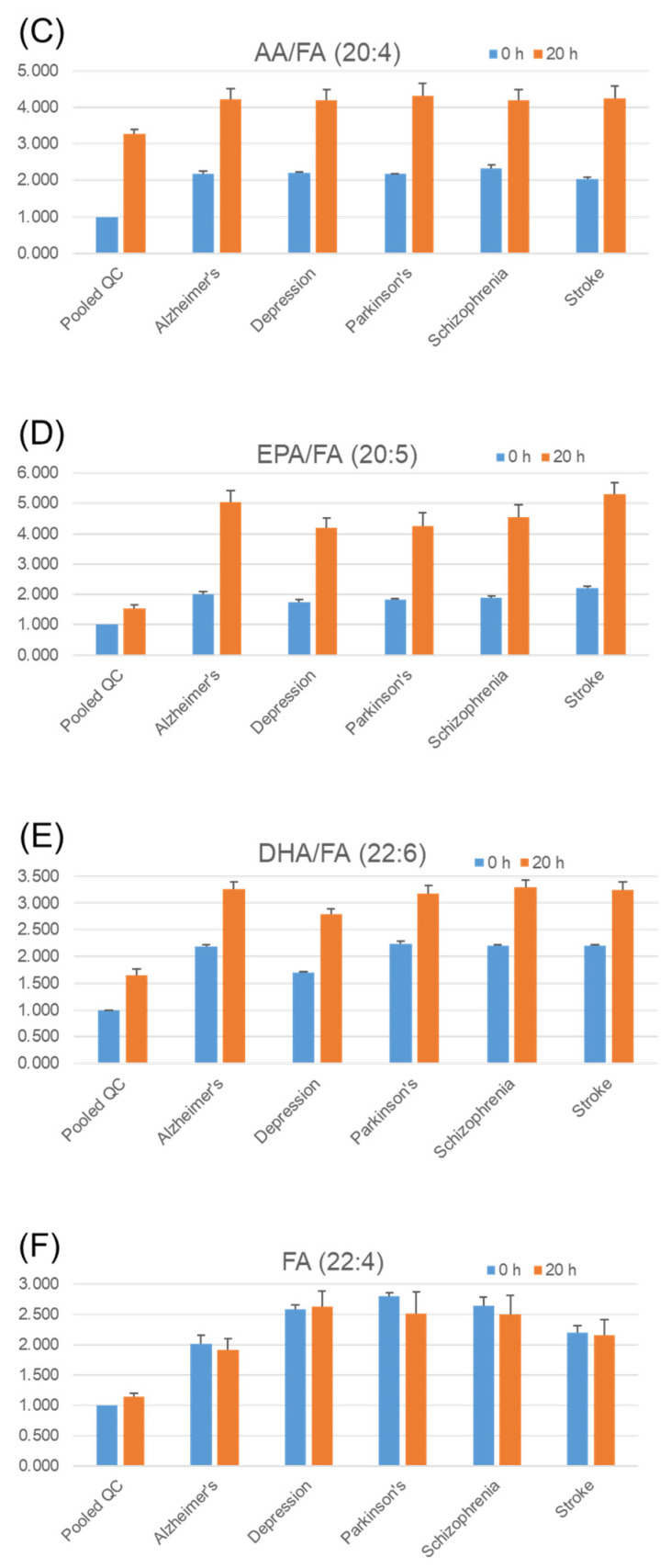
Stability of endogenous free fatty acids in serum. Pooled quality control (QC) serum samples and pooled disease serum samples were incubated at 37 °C for 20 h. Three replicates of each pooled serum sample were tested. Extraction, tandem mass tag (TMT) derivatization, and LC/MS analysis were performed as described in the Methods section. The *Y*-axis shows the values relative to those of pooled QC serum with no incubation (0 h) for each lipid. Error bars represent the standard error of the mean. FA, fatty acid; AA, arachidonic acid; EPA, eicosapentaenoic acid; DHA, docosahexaenoic acid. (**A**) FA (18:1), (**B**) FA (20:1), (**C**) AA/FA (20:4), (**D**) EPA/FA (20:5), (**E**) DHA/FA (22:6), (**F**) FA (22:4).

The usefulness of serum lipid analysis has already been reported with respect to several aspects [30,31]. To screen the trends in the difference in the stability of serum lipids in a variety of diseases, we tested a pooled serum mixture of the same amount of individual serum from patients with each disease. We also randomly selected 300 serum samples as pooled QC samples among approximately 6000 patients (average age: ~60, female: ~50%) who visited the University of Tokyo Hospital for disease treatment and diagnosis. We performed TMT labeling of six different reporter ions based on six different pooled serum samples from a total of five diseases and a QC sample, that is, a simultaneous comparison of six different TMT-labeled samples in a single LC/MS run. To test the stability of serum lipids, we used an incubation temperature of 37 °C, which is similar to that inside the body. The level relative to that of the QC pooled serum and the stability of endogenous eicosanoids, fatty acids, and LPA in each disease serum sample are shown in Figure 5. Regarding free fatty acid (FA), FA (18:1), and FA (20:1) (Figure 6A,B), the level between before and after incubation did not seem to vary significantly among diseases, whereas arachidonic acid (AA, FA (20:4)) increased in all six samples (Figure 6C), and a similar trend was observed for eicosapentaenoic acid (EPA, FA (20:5)) (Figure 6D) and docosahexaenoic acid (DHA, FA (22:6)) (Figure 6E), whereas FA (22:4) did not change significantly (Figure 6F).

**Figure 7:**
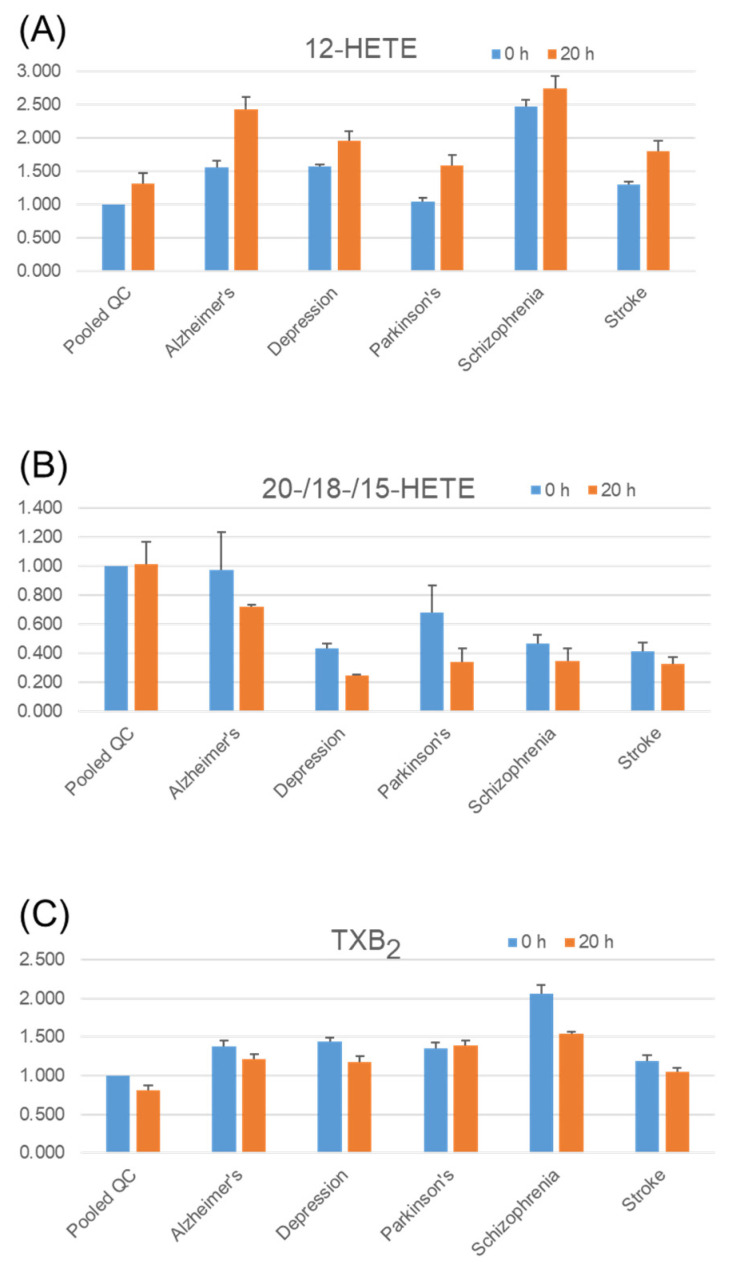

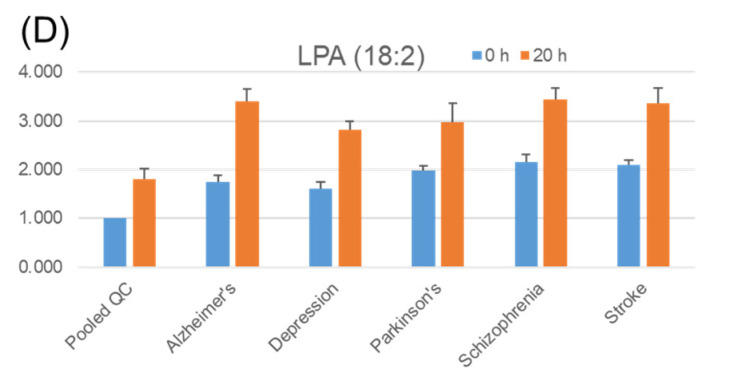
Stability of endogenous eicosanoids and LPA in serum. Pooled quality control (QC) serum samples and pooled disease serum samples were incubated at 37 °C for 20 h. Three replicates of each pooled serum sample were tested. Extraction, tandem mass tag (TMT) derivatization, and LC/MS analysis were performed as described in the Methods section. The *Y*-axis shows the values relative to those of pooled QC serum with no incubation (0 h) for each lipid. Error bars represent the standard error of the mean (SEM). HETE, hydroxyeicosatetraenoic acid; TXB2, thromboxane B2, LPA, lysophosphatidic acid. (**A**) 12-HETE, (**B**) mixture of 20-HETE/18-HETE/15-HETE, or one of them, (**C**) TXB_2_, (**D**) LPA (18:2).

Despite the approximate chemical structure, the cause of the differences in stability among the different molecular species is not likely to be a simple oxidation or light-induced reaction. Group 10 secretory phospholipase A2 (PLA2G10) is known to hydrolyze phospholipids and shows an apparent preference for PUFAs, such as EPA, DPA, and AA, with respect to substrate phospholipids [32]. The results indicate that PLA2G10 might be activated in freeze-thawed serum. For eicosanoids, 12-HETE (hydroxyeicosatetraenoic acid) tended to increase (Figure 7A) after incubation, for example, whereas 20-/18-/15-HETE tended to decrease in serum levels (Figure 7B). Since 12-HETE and 15-HETE are downstream of lipoxygenase enzymes and 18-HETE and 20-HETE are produced by a cytochrome P450 enzyme, it is not surprising that the activity of these enzymes is influenced by diseases [33]. In disease samples, thromboxane B_2_ (TXB_2_) tended to decrease in serum after incubation, especially for patients with schizophrenia (Figure 7C). One type of molecule that appears to play an important role in this disease is eicosanoids such as TXB_2_ [34]. As already reported in papers on LPA [23,29,35], LPA (18:2) was observed in serum and it was increased by incubation (Figure 7D).

**Figure 8:**
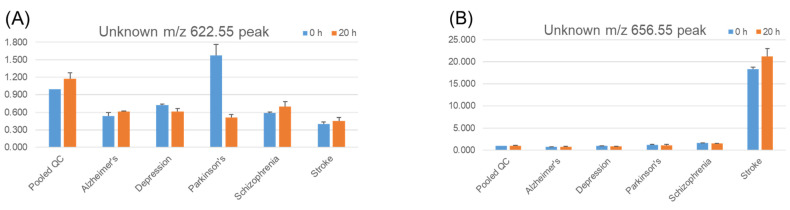
Stability of endogenous unknowns in serum. Pooled quality control (QC) serum samples and pooled disease serum samples were incubated at 37 °C for 20 h. Three replicates of each pooled serum sample were tested. Extraction, tandem mass tag (TMT) derivatization, and LC/MS analysis were performed as described in the Methods section. The *Y*-axis shows the values relative to those of pooled QC serum with no incubation (0 h) for each lipid. Error bars represent the standard error of the mean (SEM). (**A**) Unknown peak at *m*/*z* 622.55, (**B**) Unknown peak at *m*/*z* 656.55.

We detected a number of unknown peaks with TMT labels that could not be identified as molecular species. Representative examples of their stability in serum are shown in Figure 8. The unknown *m*/*z* 622.55 peak showed a decrease in serum levels for the pooled Parkinson’s disease sample (Figure 8A), but others did not show such reductions. Apart from serum stability, an unknown *m*/*z* 656.55 peak (Figure 8B) was detected as a higher signal in stroke patient serum among other diseases. The unknown *m*/*z* 622.55 peak was set up as the monitoring channel for HETEs and epoxyeicosatrienoic acids (EETs), eicosanoid subcategories with number of structural isomers (i.e., 5-HETE, 8-HETE, and 11-HETE, among others, for HETEs or 5(6)-EET, 8(9)-EET, and 14(15)-EET, among others, for EET). The unknown *m*/*z* 656.55 peak corresponded to prostaglandins D_1_ and E_1_, prostaglandin F_2_, and their isomers. Because the standard compounds derivatized with TMT were not fully separated from each other, we did not confirm these peaks as one of these candidates.

It might be possible to isolate all eicosanoids that are the same or close in molecular weight and identify each peak with very sophisticated HPLC conditions. However, in many cases, HPLC separation is a time-consuming procedure that conflicts with high-throughput multi-specimen analysis, which is characteristic of the TMT approach. Therefore, if an interesting candidate peak is found by high-throughput multi-specimen analysis, it is followed by another analytical method focusing on that peak for compound identification. Apart from peak identification, the stability and expression levels of these substances in the serum might differ depending on the type of lipid or disease. In this study, we developed a multi-specimen analysis for serum lipids and a relative quantification method using a pooled QC sample with TMT labeling. Stability of the lipids in human serum samples were examined. Stability was different among lipid species and also among samples, and we were reminded of the importance of process control from blood collection to serum preparation and storage.

## 3. Materials and Methods

### 3.1. Reagents

AminoxyTMT sixplex Label Reagent Set was purchased from Thermo Fisher Scientific Inc. (Waltham, MA, USA). Phospholipid mixtures were obtained from Avanti Polar Lipids, Inc. (Alabaster, AL, USA). Lipid mediator standards, 12-HETE, 20-HETE, TXB_2_, PGE_2_, arachidonic acid, tetranor-PGDM, and 20-carboxy-LTB_4_ were obtained from Cayman Chemical (Ann Arbor, MI, USA). LPA (16:0), LPA (18:1), and PA (36:2) were obtained from Avanti Polar Lipids (Alabaster, AL, USA). DMTMM and 4-methylmorpholine (NMM) were purchased from Sigma-Aldrich Co. (St. Louis, MO, USA). 1-Ethyl-3-[3-dimethylaminopropyl]carbodiimide hydrochloride (EDC) was obtained from Fuji Film Wako Pure Chemical Co. (Osaka, Japan). Special grade reagents of ammonium bicarbonate, sodium chloride and 1-buthanol, and HPLC grade solvents of methanol, isopropanol and acetonitrile were purchased from Wako Pure Chemical Co. The authentic lipid standard stock solutions were 12-HETE (0.6 μg/mL), 20-HETE (0.6 μg/mL), TXB_2_ (0.6 μg/mL), PGE_2_ (0.6 μg/mL), AA (0.6 μg/mL), tetranor-PGDM (0.6 μg/mL), 20-carboxy-LTB_4_ (0.6 μg/mL), LPA(16:0) (10 μM), LPA (18:1) (10 μM), PA (36:2) (5 μg/mL).

### 3.2. Samples

This study was conducted with the approval of the ethics committee of the University of Tokyo Hospital in compliance with the relevant guidelines and regulations. A QC sample was generated by pooling 300 patient residual samples after the completion of the requested clinical laboratory tests at the University of Tokyo Hospital (the approval number by the Institutional Research Ethics Committee of the Faculty of Medicine, University of Tokyo, was 2019022NI). The Japanese neurodegenerative and psychiatric disease specimens were obtained from residue samples from patients who had been examined at the University of Tokyo Hospital during a 3-week period (the approval number by the Institutional Research Ethics Committee of the Faculty of Medicine, The University of Tokyo, was 2019068NI). Informed consent for participation in the study was obtained using an opt-out process on the web page of The University of Tokyo Hospital. The investigations were carried out following the rules of the Declaration of Helsinki of 1975 (https://www.wma.net/what-we-do/medical-ethics/declaration-of-helsinki/), revised in 2013. Diagnosis in the electronic medical record system recorded within 2 years before the date of blood sampling was used. All samples were stored at −80 °C prior to analysis.

### 3.3. Lipid Extraction

We added 15 µL of 2 M NaCl to 30 µL of serum and mixed it well. Next, 240 µL of 1-butanol was added to the sample, mixed, and then centrifuged at 10,000× *g* for 2 min at 4 °C, and 200 µL of the supernatant was transferred into a new tube, and the solvent was allowed to evaporate using a vacuum evaporator (EC-95C3T, SAKUMA, Tokyo, Japan). In the experiment on extraction, the results of LC/MS analysis without extraction were compared with the results after extraction, but in this case, the comparison with the addition of standards, but without TMT derivatization.

### 3.4. TMT Reaction

The samples after extraction and drying were resuspended in 20 µL methanol in the tube. Then, 20 µL of 1% NMM in chloroform was added and mixed. Next, 10 µL of 33.3 mg/mL EDC in methanol was added to the sample tube and 40 µL of aminoxyTMT dissolved in 200 µL of acetonitrile was mixed. Then, 10 µL of 10 mg/mL DMTMM in methanol was added and the mixture was allowed to react at room temperature for 24 h. Finally, 5 µL of 10% acetone in methanol was added to quench the unreacted reagent. After completion of TMT derivatization, equal amounts were mixed to form one set of six TMT reporter ions (numbers 126–131) to make one vial.

### 3.5. Liquid Chromatography/Mass Spectrometry (LC/MS) Measurements

The LC and MS measurement conditions were as follows. The sixplex aminoxyTMT™ derivatized samples were subjected to the Nexera UHPLC system and LCMS-8040 or -8060 triple quadrupole mass spectrometers (Shimadzu Co., Kyoto, Japan). The aminoxyTMT derivatized samples were diluted twice with methanol before measurement. An Acquity UPLC BEH C8 column (1.7 μm, 2.1 × 100 mm; Waters) was used with the following mobile phase compositions: 5 mM NH4HCO3/water (mobile phase A), acetonitrile (mobile phase B), and isopropanol (mobile phase C). The pump gradient was programmed as follows [time (%A/%B/%C)]: 0 min (95/5/0), to 8 min (70/30/0), 16 min (30/35/35), 28 min (6/47/47), 35 min (6/47/47), 35.1 min (95/5/0); it was then held for 38 min for equilibration. The flow rate was 0.35 mL/min, and column temperature was 47 °C. The injection volume was 5 μL. SRM analysis was performed using the positive ion mode ESI, with a collision energy of 46 eV for TMT-derivatized compounds. For the sixplex aminoxyTMT sample, Fatty acid species possessing carbon chains 12 to 24 carbons in length were targeted [22], 132 eicosanoids or other fatty acid derivatives [36], five LPA species (16:0, 18:0, 18:1, 18:2, 20:4), and nine PA species (32:0, 34:1, 34:2, 36:0, 36:2, 36:3, 36:2, 36:4, 38:4) were targeted. The selective reaction monitoring (SRM) transitions for TMT-derivatized molecules, [M-H+303.25]+ →126.15, →127.15, →128.15, →129.15, →130.15, and →131.15 were used. The SRM transitions for LPC (16:0), LPE (18:1), and DAG (18:1/18:1), 496.35 → 184.1, 480.3 → 339.3, and 636.6 → 339.3, respectively, in positive ion mode. The SRM transitions for non-labeled LPA (16:0), LPA (18:1), and PA (36:2), were 409.4 → 153.1, 435.4 → 153.1, and 699.6 → 153.1, respectively, in negative ion mode. The SRM settings for non-labeled eicosanoids have been described previously [36]. All data were analyzed using Microsoft Excel 2016.

## 4. Conclusions

In the TMT method, six samples are mixed into one and analyzed by LC/MS as a single set. Since six LC/MS measurements can be completed in a single run, a six-fold increase in sample throughput can be achieved compared to conventional methods. This is one of the merits, and another advantage is quantitation. The TMT method does not require internal standards, which have to be obtained and prepared depending on the number of targets. Instead, a common bridging sample is prepared to normalize data. One of the six samples is a bridging sample (pooled QC sample) that is common among the sets. Since we perform relative quantification to this bridging sample, we can obtain reliable quantification values as long as we use the bridging sample. We have made it possible to derivatize lipid molecules with carboxy groups and lipid molecules with phosphate groups at the lipid ends simultaneously by TMT. However, there are many lipid molecules that cannot be TMT-derivatized. Therefore, it is necessary to develop a new TMT method to expand the range of application. We mixed 300 individual serum samples as a pooled QC sample to make a common bridging sample. The disadvantage of this method is that relative quantification is not possible for peaks that are not detected in the pooled QC samples.

In serum preparations, blood samples are kept at room temperature for 30–60 min after collection to allow clots to form and remove cells, clotting factors, and other cellular components. It is easy to imagine from our results that the inadequate control of temperature and time in this step would lead to large variations in data [37,38,39]. Therefore, proper sample collection, preparation, and storage are important to obtain reliable outcomes. However, the association between the disease and serum lipid stability is of great interest because various factors, such as enzyme activity affected by the disease situation in serum, might affect the stability.

We believe that the method developed here can be applied to the analysis of many clinical samples, not only for serum samples but also organ samples and various cell samples. The TMT labeling method is expected to be one of the most useful analytical methods for bioactive lipids and lipidomics.

## Figures and Tables

**Table 1:** Reproducibility of peak area obtained by tandem mass tag (TMT) derivatization methodology in different concentrations of standard lipids.

	**12-HETE × 16**	**12-HETE × 8**	**12-HETE × 4**	**12-HETE × 2**	**12-HETE × 1**	**Correlation**
****CV% in Buffer****	8.04	13.03	13.36	4.42	5.26	0.966
**CV% in Serum**	4.18	8.23	4.60	2.93	1.78	0.951
	**20-HETE × 16**	**20-HETE × 8**	**20-HETE × 4**	**20-HETE × 2**	**20-HETE × 1**	**Correlation**
**CV% in Buffer**	6.86	8.51	11.01	5.79	4.03	0.963
**CV% in Serum**	4.01	8.54	3.01	2.71	2.99	0.949
	**PGE**_**2**_ ** × 16**	**PGE**_**2**_ ** × 8**	**PGE**_**2**_ ** × 4**	**PGE**_**2**_ ** × 2**	**PGE**_**2**_ ** × 1**	**Correlation**
**CV% in Buffer**	5.14	8.97	8.20	4.34	5.91	0.962
**CV% in Serum**	4.01	8.54	3.01	2.71	2.99	0.949
	**TXB**_**2**_ ** × 16**	**TXB**_**2**_ ** × 8**	**TXB**_**2**_ ** × 4**	**TXB**_**2**_ ** × 2**	**TXB**_**2**_ ** × 1**	**Correlation**
**CV% in Buffer**	7.93	10.18	12.41	4.91	6.14	0.999
**CV% in Serum**	4.81	6.65	5.86	7.51	7.75	0.998
	**20-carboxy-LTB**_**4**_ ** × 16**	**20-carboxy-LTB**_**4**_ ** × 8**	**20-carboxy-LTB**_**4**_ ** × 4**	**20-carboxy-LTB**_**4**_ ** × 2**	**20-carboxy-LTB**_**4**_ ** × 1**	**Correlation**
**CV% in Buffer**	11.30	10.59	27.28	14.91	12.27	0.994
**CV% in Serum**	15.26	6.23	5.42	5.70	11.09	0.996
	**tetranor-PGDM × 16**	**tetranor-PGDM × 8**	**tetranor-PGDM × 4**	**tetranor-PGDM × 2**	**tetranor-PGDM × 1**	**Correlation**
**CV% in Buffer**	18.83	14.52	28.35	14.87	12.62	0.996
**CV% in Serum**	6.69	9.37	7.06	9.13	10.40	0.978
	**LPA (16:0) × 16**	**LPA (16:0) × 8**	**LPA (16:0) × 4**	**LPA (16:0) × 2**	**LPA (16:0) × 1**	**Correlation**
**CV% in Buffer**	22.15	17.68	16.81	16.69	8.02	0.998
**CV% in Serum**	8.40	9.57	9.13	10.64	2.79	0.998
	**LPA (18:1) × 16**	**LPA (18:1) × 8**	**LPA (18:1) × 4**	**LPA (18:1) × 2**	**LPA (18:1) × 1**	**Correlation**
**CV% in Buffer**	16.32	15.21	19.61	9.43	7.52	0.998
**CV% in Serum**	6.18	9.55	12.70	7.47	4.79	0.989
	**PA (36:2) × 16**	**PA (36:2) × 8**	**PA (36:2) × 4**	**PA (36:2) × 2**	**PA (36:2) × 1**	**Correlation**
**CV% in Buffer**	-	12.29	12.55	5.27	4.03	0.999
**CV% in Serum**	2.55	7.21	5.23	5.22	2.02	0.995

Standard lipids were added to PBS buffer (*n* = 3) or human serum (*n* = 4). The final concentration in ×1 sample was 170 ng/mL for 12-hydroxyeicosatetraenoic acid (12-HETE), 20-hydroxyeicosatetraenoic acid (20-HETE), thromboxane B_2_ (TXB_2_), prostaglandin E_2_ (PGE_2_), arachidonic acid (AA), tetranor-PGDM, and 20-carboxy-LTB4, 0.9 μM for lysophosphatidic acid (LPA)(16:0) and LPA(18:1), and 0.47 µg/mL for phosphatidic acid (PA)(18:1/18:1). Serial 2-fold dilutions of standard mixtures were performed to prepare ×2, ×4, ×8, and ×16 diluted standards. The coefficient of variation (CV) for area value in each peak area of PBS buffer (*n* = 3) or human serum (*n* = 4) samples was calculated. The correlation coefficient between the dilution factor and peak area was calculated using the CORREL function add-in in Excel 2016.

**Table 2:** Demographic characteristics of patients.

	Alzheimer’s Disease	Depression	Parkinson’s Disease	Schizophrenia	Stroke
**N**	67	26	21	21	37
**Female %**	62.7%	53.8%	52.4%	42.9%	37.8%
**Age (years) mean ± SD**	80.3 ± 7.18	75.3 ± 6.70	74.4 ± 5.50	72.7 ± 5.82	77.5 ± 6.07
**Age (years) Range**	62–95	65–88	66–90	65–82	65–90

## Data Availability

The data presented in this study are available in article.
